# Comparative Study for Spectrofluorimetric Determination of Ambroxol Hydrochloride Using Aluminum Metal Transfer Chelation Complex and Biogenic Synthesis of Aluminum Oxide Nanoparticles Using *Lavandula spica* Flowers Extract

**DOI:** 10.3390/molecules28052210

**Published:** 2023-02-27

**Authors:** Najla S. Al-Humud, Salma A. Al-Tamimi, Amal M. Al-Mohaimeed, Maha F. El-Tohamy

**Affiliations:** Department of Chemistry, College of Science, King Saud University, P.O. Box 22452, Riyadh 11495, Saudi Arabia

**Keywords:** ambroxol hydrochloride, metal chelation, charge transfer, *Lavandula spica* flowers, nanomaterials

## Abstract

The existing study pronounces two newly developed spectrofluorimetric probes for the assay of ambroxol hydrochloride in its authentic and commercial formulations using an aluminum chelating complex and a biogenically mediated and synthesized aluminum oxide nanoparticles (Al_2_O_3_NPs) from *Lavandula spica* flower extract. The first probe is based on the formation of an aluminum charge transfer complex. However, the second probe is based on the effect of the unique optical characteristics of Al_2_O_3_NPs in the enhancement of fluorescence detection. The biogenically synthesized Al_2_O_3_NPs were confirmed using various spectroscopic and microscopic investigations. The fluorescence detections in the two probes were measured at a λ_ex_ of 260 and 244 and a λ_em_ of 460 and 369 nm for the two suggested probes, respectively. The findings showed that the fluorescence intensity (FI) covered linear concentration ranges of 0.1–200 ng mL^−1^ and 1.0–100 ng mL^−1^ with a regression of ˃0.999 for AMH-Al_2_O_3_NPs-SDS and AMH-Al(NO_3_)_3_-SDS, respectively. The lower detection and quantification limits were evaluated and found to be 0.04 and 0.1 ng mL^−1^ and 0.7 and 0.1 ng/mL^−1^ for the abovementioned fluorescence probes, respectively. The two suggested probes were successfully applied for the assay of ambroxol hydrochloride (AMH) with excellent percentage recoveries of 99.65% and 99.85%, respectively. Excipients such as glycerol and benzoic acid used as additives in pharmaceutical preparations, several common cations, and amino acids, as well as sugars, were all found to have no interference with the approach.

## 1. Introduction

Lung diseases, often known as respiratory disorders, include tuberculosis, lung cancer, mesothelioma, cystic fibrosis, asthma, and cystic fibrosis. Lung disease can cause serious health issues, troubling symptoms, and potentially fatal situations if it is not treated [1]. Mycobacterium tuberculosis (TB) affects around one-third of the global population, and it causes 2 million deaths annually. According to the National Institutes of Health, TB kills more people than any other disease brought on by a single infectious agent [2].

The drug ambroxol hydrochloride (AMH) is a member of the group of medications known as “mucolytic agents” (cough/sputum thinner), and it is primarily used to treat acute (short-term) and chronic (long-term) respiratory disorders characterized by an excess of mucus. Laryngitis, tracheitis, acute and recurrent bronchitis (infection of the airways), and chronic disorders, including chronic bronchitis and chronic obstructive pulmonary disease, are all treated with ambroxol hydrochloride [3].

A careful survey of the literature revealed the presence of various techniques that have been addressed for the assay of AMH in different matrices, including bulk powder, dosage forms, and biosamples. Among these is the simultaneous determination of AMH in combination with other drugs using chromatographic separation methods [4,5]. Al Syed et al. described facile spectrofluorimetric probes for the estimation of AMH as an active metabolite of bromhexine hydrochloride [6]. Other spectrophotometric methods have been suggested for the determination of AMH in its pharmaceuticals and biological fluids [7,8,9]. Few electrochemical approaches for the determination of AMH have been reported, including voltametric and wireless sensors [10,11]. The majority of the techniques described in the literature possess certain limitations, particularly chromatographic separation, such as lack of a universal detector, low separation efficiency, time-consuming, and require large quantities of solvents [12]. Fluorescence spectroscopy is a desirable alternative technique due to its intrinsic sensitivity and ease in spectral acquisition, in which little sample preparation is required [13]. By advancing attention to using more sensitive materials, attempts have been intensive in using different components, such as metal chelation and nanomaterials [14]. The presence of dibromobenzylamino moiety in the ambroxol hydrochloride molecule has been reported to have a native fluorescence (FL) behavior in the compound. However, measuring the FL of the drug exhibited a very weak native value. The presence of the amino group in the drug and oxygen group in the reagent aluminum nitrate facilitates the ability of metal chelate with excellent stability [15]. The toughest trivalent metal ion (Al) is very electropositive and does not polarize easily. The interaction between aluminum and its ligands is noncovalent and typically involves ionic or electrostatic bonding. Al loves to coordinate with hard Lewis bases that provide electrons to its empty electron orbitals, such as OH^−^, F^−^, PO_4_^3−^, SO_4_^2−^, CH_3_COO^−^, ROH, RO^−^, and RNH_2_. Al complexes with multidentate ligands that are negative oxygen donors are the most stable [16].

Nanomaterials are highly sought after in numerous scientific disciplines, and metal oxides in the construction of fluorescent hybrid materials, in particular, have aroused a lot of interest [17]. Among these nanomaterials are metal oxides such as ZnO, CuO, NiO, and Al_2_O_3_ nanoparticles. The Al_2_O_3_ nanoparticles find their purposes in almost all areas, such as material engineering, drug targeting systems, catalysis, and biomedicals [18,19,20,21]. Physical and chemical processes can be used in a number of ways to create nanomaterials [22]. The findings indicated that these procedures have a number of disadvantages, including the need for expensive and toxic stabilizers or capping agents, toxic organic solvents or hazardous materials, higher temperatures required for final product preparation, and more [23]. Due to this, researchers are interested in developing green methods for the synthesis of nanomaterials [24].

Lavender is an important medicinal plant of the Lamiaceae family. It is indigenous to the Mediterranean region and grows naturally in the lower regions of mountains. Many countries in Europe, North Africa, Southwest Asia, western Iran, eastern India, China, and Japan grow lavender as a decorative plant. *Lavandula spica* is one of the species in the Lavandula genus in which the main active constituents of its lavender oil are linalool, linalyl acetate, terpinen-4-ol, and camphor. The phytochemicals in *Lavandula spica* can act as reducing, capping, and stabilizing agents for the biogenic synthesis of nanomaterials [25,26].

The objective of this work was to develop new ultrasensitive spectrofluorimetric approaches based on metal chelation with aluminum nitrate and green synthesized, mediated *Lavandula spica* flower extract Al_2_O_3_ nanoparticles for the estimation of ambroxol hydrochloride in its authentic and commercial syrup. The effective conditions of the two suggested approaches were investigated and optimized. The validity of both systems was proved, and a comparative study was performed between the results of the two developed methods.

## 2. Results and Discussion

### 2.1. Characterization of Green Synthesized Al_2_O_3_NPs

**Figure 1:**
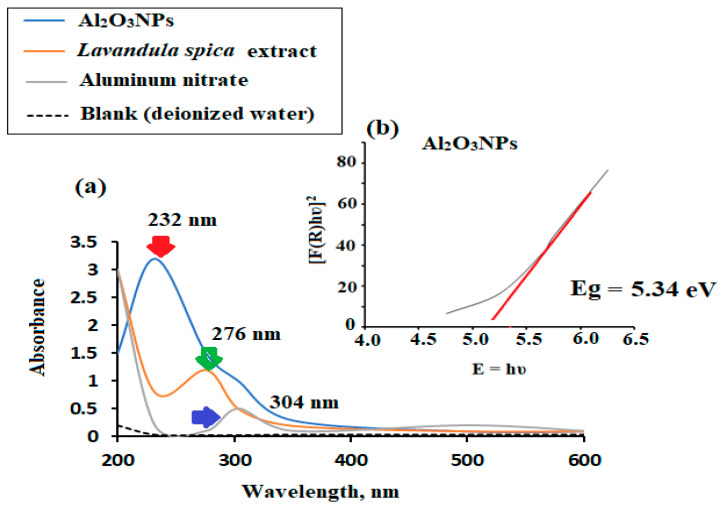
(**a**) UV-vis spectra of Al_2_O_3_NPs, *Lavandula spica* extract, aluminum nitrate, and blank (deionized) water and (**b**) the band gap of Al_2_O_3_NPs.

UV-vis spectroscopy is a great method for identifying, characterizing, and investigating nanoparticles since their optical properties are sensitive to size, shape, concentration, aggregation state, and refractive index near the nanoparticle surface. The UV-vis spectra of Al_2_O_3_NPs, *Lavandula spica* flowers extract, and aluminum nitrate showed the presence of three distinct peaks at 232, 276, and 304 nm for the abovementioned samples (Figure 1a). Several parameters, including band gap, oxygen deficit, surface roughness, and impurity centers, are expected to influence absorbance [27]. The absorption spectrum of Al_2_O_3_NPs exhibited at 232 nm can be resulted from the photoexcitation of electrons from the valence to the conduction band. To calculate the direct band gap of Al_2_O_3_NPs, a Tauc plot was carried out, and the band gap was estimated from the following energy quantum mechanics Equation (1):

Eg = hC/λ(1)

where the band gap, blank constant (6.626 × 10^−34^ J.s), velocity of light (2.99 × 10^8^ m/s), and absorption band are expressed as Eg, h, C, and λ, respectively. The estimated band gap of presynthesized Al_2_O_3_NPs was found to be 5.34 eV. The obtained optical behavior results of Al_2_O_3_NPs were in agreement with previously reported values in the literature [28].

**Figure 2:**
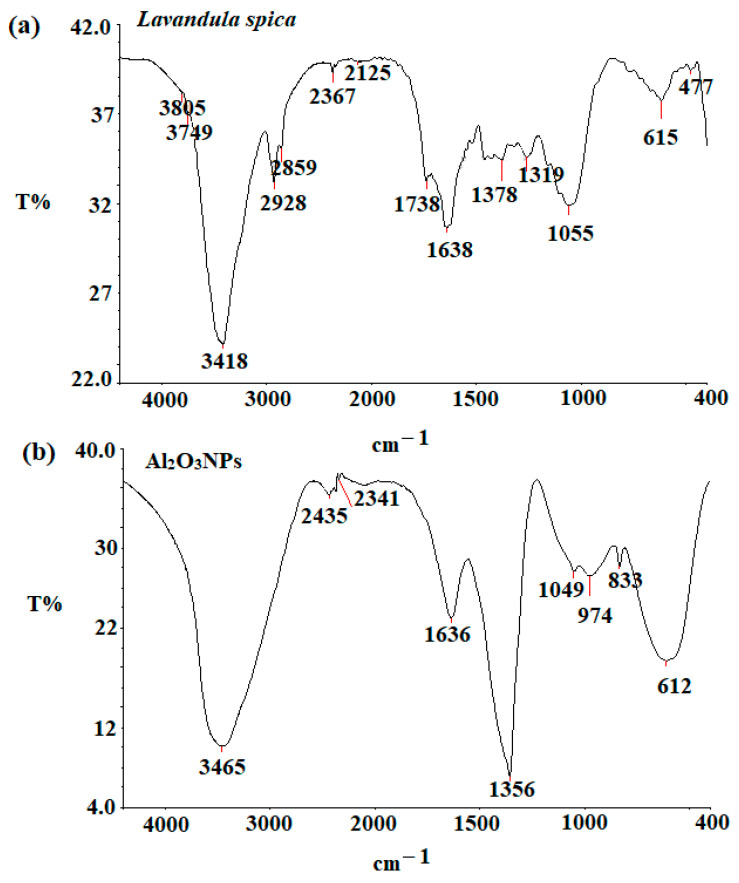
FT-IR spectra of (**a**) *Lavandula spica* extract and (**b**) Al_2_O_3_NPs measured at 4400–400 cm^−1^.

FT-IR measurement was performed to determine the functional groups responsible for the reduction of aluminum nitrate to Al_2_O_3_NPs and their stability. The investigation of any probable oxidation of the extract was carried out using FT-IR analysis because the reduction of the aluminum nitrate ions should be connected with the oxidation of the *Lavandula spica* extract. The FT-IR spectrum of *Lavandula spica* extract (Figure 2a) showed three absorption peaks at 3705, 3749, and 3418 cm^−1^, corresponding to medium, sharp, and broad O-H stretching vibration of free and intermolecular bonded alcohol, respectively. The absorption peaks at 2367, 1738, 1638, 1378, and 1055 cm^−1^ represent the presence of O=C=O stretching of carbon dioxide, strong C=O stretching vibration of ester, strong C=C stretching of alkene, O-H bending vibration of phenol, and strong C-O stretching of a primary alcohol, respectively [29]. However, the FT-IR analysis of Al_2_O_3_NPs (Figure 2b) displayed nine remarkable peaks at 3465 (O-H stretching vibration of intermolecular bonding of alcohol), 2435, and 2341 cm^−1^ (O=C=O stretching of carbon dioxide), 1636 (strong C=O stretching vibration of ester), 1356 (medium O-H bending of carboxylic acid), 1049 (strong C-O stretching of primary alcohol), 974 and 833 (strong C-H bending of the trisubstituted compound), and 612 cm^−1^, corresponding to Al-O, respectively [30]. 

**Figure 3:**
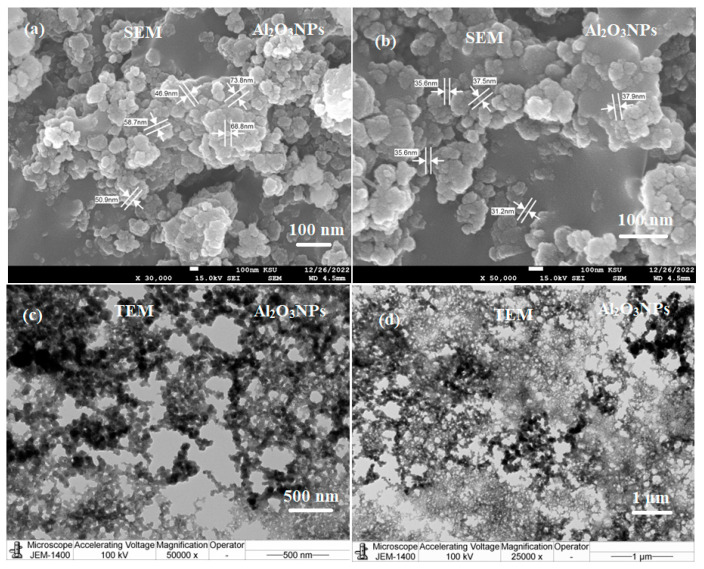
(**a**,**b**) SEM and (**c**,**d**) TEM images of Al_2_O_3_NPs synthesized using *Lavandula spica* flower extract measured at different magnifications.

Furthermore, morphology and size were determined using typical microscopic techniques such as scanning electron microscopy (SEM) and transmission electron microscopy (TEM), while elemental analysis was determined using energy-dispersive X-ray spectroscopy (EDX). The SEM images (Figure 3a,b) revealed a fine particulate matter with elongated, spherical particles that appear to be an aggregation of distinct cotton form structures. In contrast to the homogeneous arrangement, spherical-shaped crystals were detected.

TEM micrographs (Figure 3c,d) showed spherical-shaped particles with an average particle size ranging from 30–75 nm at different magnifications. However, other larger aggregate-formed molecules were observed to have sizes of 100 nm [30].

**Figure 4:**
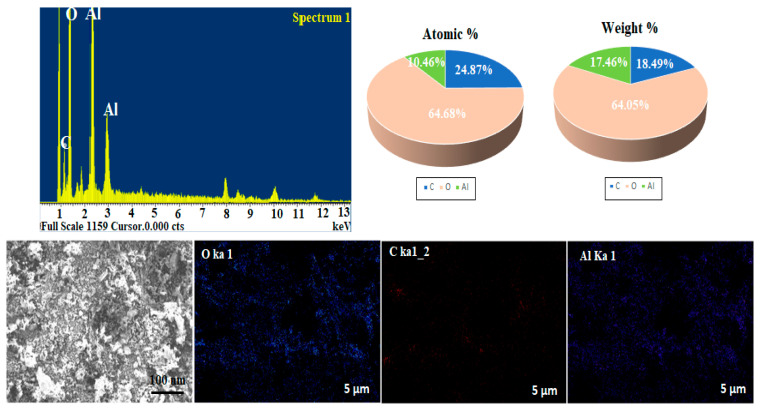
EDX and mapping of Al_2_O_3_NPs synthesized using *Lavandula spica* flower extract.

The elemental analysis of the green synthesized Al_2_O_3_NPs prepared by *Lavandula spica* flowers extract was carried out using SEM in conjunction with an EDX spectrometer. According to the obtained results, it was found that in the Al_2_O_3_NPs sample, the weight% of Al, C, and O was 17.46%, 18.49%, and 64.05%, while the atomic% was recorded to be 10.46%, 24.78%, and 64.68%. EDX mapping of the as-prepared Al_2_O_3_NPs was demonstrated, and the outcomes of SEM, EDX, and elemental mapping confirmed the successful formation of Al_2_O_3_NPs (Figure 4).

### 2.2. Spectral Characteristics

Fluorescence of spectrofluorimetry is an essential analytical tool in chemistry and material science. It plays an important role in pharmaceutical determinations with low concentrations, in which high-throughput fluorescence measurements are carried out at less than µg mL^−1^.

**Figure 5:**
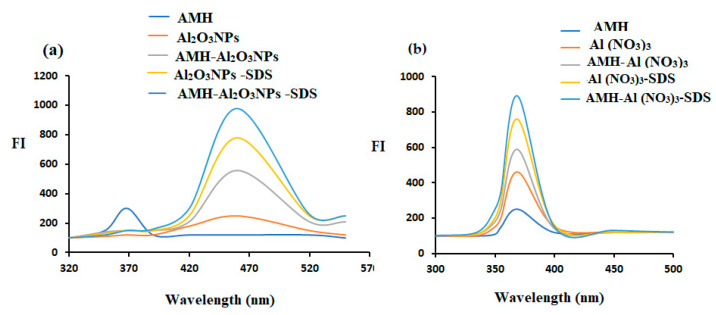
Fluorescence spectra of 1.0 µg mL^−1^ of AMH solution with (**a**) Al_2_O_3_NPs (**b**) and Al (NO_3_)_3_ in the presence of SDS.

Probe 1: AMH was found to exhibit an emission band of weak strength at λ_ex_ = 260 and λ_em_ 460 nm. The addition of Al_2_O_3_NPs to the FL system enhanced the emission bands in the FL system (Figure 5a). Moreover, FL analysis could be successfully used to determine the investigated drug.

Probe 2: Following excitation at 244 nm, it was observed that the AMH had a weakly excitation-induced emission band at 369 nm. Each FI system’s emission bands were improved by the inclusion of Al(NO_3_)_3_ and SDS. This can be attributed to the ability of Al (III) to form fluorogenic ligands with poorly native fluorescence compounds [31]. The tested drug could also be successfully quantified using FI analysis (Figure 5b).

### 2.3. Optimization of Analytical Conditions

The experimental conditions of the proposed FL technique were optimized with respect to various parameters, including the effect of solvents, volume of Al_2_O_3_NPs and Al(NO_3_)_3_ solutions, buffer, volume of buffer, pH, response time, surfactant, and volume of SDS (Table 1).

### 2.4. Effect of Solvent

**Figure 6:**
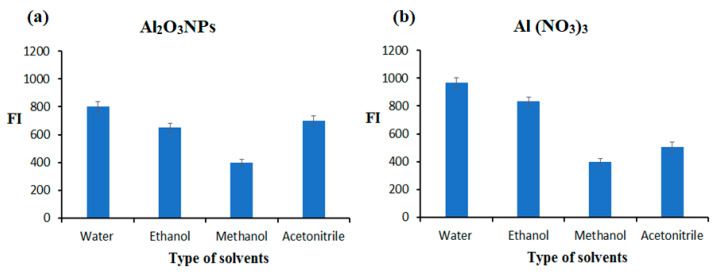
Effect of solvents on the FI of 1.0 µg mL^−1^ AMH soluble in various solvents in the absence of (**a**) metal oxide nanoparticles (Al_2_O_3_NPs) and (**b**) metal-chelating agent (Al(NO_3_)_3_).

The influence of various types of solvents (water, ethanol, methanol, and acetonitrile) on the FI spectrum of the AMH solution (1.0 µg mL^−1^) was tested. The maximum FIs were measured according to the nature of the solvent. Figure 6a,b illustrates the effect of the solvents on the FI of the AMH solution at λ_ex_ and λ_em_ wavelengths 260/460 and 244/369 and 260 for the two suggested FL methods, respectively.

### 2.5. Effect of pH

**Figure 7:**
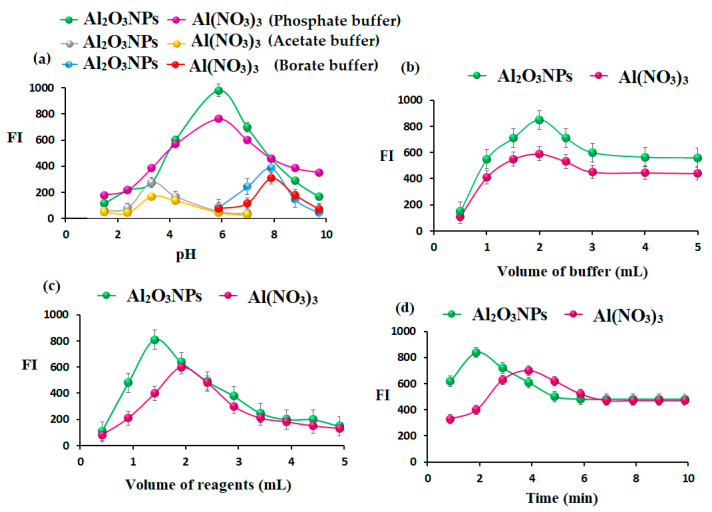
(**a**) Effect of pH using (acetate, phosphate, and borate buffers), (**b**) effect of volume of phosphate buffer pH 5.8, (**c**) effect of volume of Al_2_O_3_NPs and Al(NO_3_)_3_, and (**d**) effect of response time for the determination of AMH using the two suggested AMH-Al_2_O_3_NPs and AMH-Al(NO_3_)_3_ systems.

The FI of the AMH solutions was investigated at various pH values (3–10) using 2.0 mL of acetate (0.1 mol L^−1^), phosphate (0.2 mol L^−1^), and borate buffer (0.1 mol L^−1^) solutions. The recorded results showed that maximum FI was observed after using phosphate buffer pH 5.8 (Figure 7a). Moreover, the effect of phosphate buffer volume on the FI spectrum was tested in the range of 1.0–6.0 mL, as shown in Figure 7b. It was observed that the use of 1.5 and 2.0 mL of phosphate buffer gave the maximum FI for the determination of AMH using Al_2_O_3_NPs and Al(NO_3_)_3_, respectively.

### 2.6. Effect of Volume of Al_2_O_3_NPs and Al(NO_3_)_3_

The optimum volume of added Al_2_O_3_NPs and Al(NO_3_)_3_ in the ranges of 0.5–5.0 mL was examined using 1.0 µg mL^−1^ of AMH solution. The maximum FI peaks were recorded after adding 1.0 and 2.0 mL of Al_2_O_3_NPs and Al(NO_3_)_3_ separately to the AMH solution. Therefore, this volume was nominated as the optimum volume for further studies (Figure 7c).

### 2.7. Effect of Response Time

The impact of response time on the FI of the AMH solution in the presence of additional Al_2_O_3_NPs and Al(NO_3_)_3_ solution as metal oxide and metal-chelating agents was studied over a range of time periods (1–10 min). The obtained results showed that a quick reaction was attained after 2 min, with both suggesting FL systems (Figure 7d).

### 2.8. Effect of Surfactants

**Figure 8:**
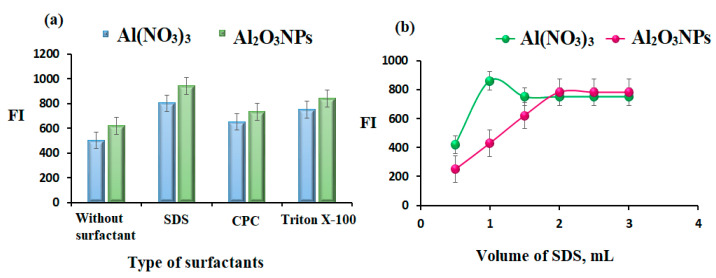
(**a**) Effect of type of surfactants and (**b**) effect of volume of SDS on the AMH 1.0 µg mL^−1^ using AMH-Al_2_O_3_NPs and AMH-Al(NO_3_)_3_ complex systems.

The FI of AMH in aqueous and different micellar media was investigated (Figure 8a) using an anionic surfactant (SDS), cationic surfactant (CPC), and nonionic surfactant (Triton X-100). All the studied organized media caused a decrease in the FI of AMH, while the SDS system gave a substantial enhancement effect on the FI of AMH in the presence of Al_2_O_3_NPs and Al(NO_3_)_3_. In addition, the influence of SDS volume on the FI of AMH was studied using different volumes of 0.01 mol L^−1^ SDS in the range of 0.5–3.0 mL. The maximum FI peaks were recorded after adding 1.0 and 2.0 mL of SDS to AMH-Al_2_O_3_NPs and AMH-Al(NO_3_)_3_ complex systems, respectively (Figure 8b).

### 2.9. Method Validation 

To ensure the suitability of the suggested analytical approach with respect to accuracy and precision for the determination of the drug under investigation, method validation was performed. The established FI systems for determining AMH were validated in accordance with the suggested ICH guidelines [32].

**Figure 9:**
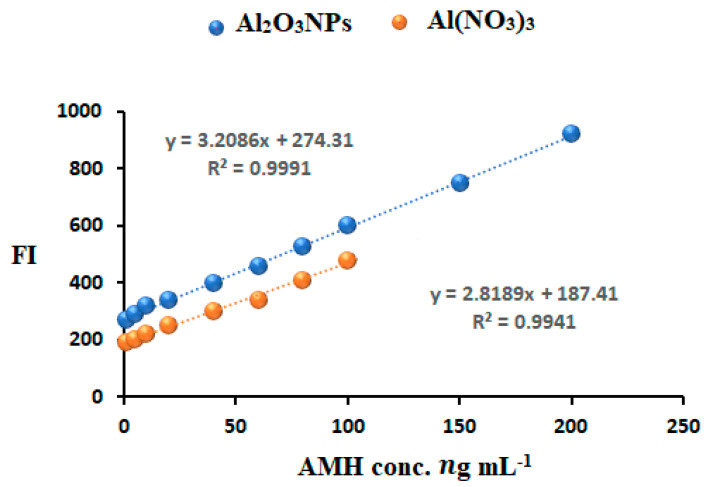
Calibration curves for AMH determination in the presence of Al_2_O_3_NPs and Al(NO_3_)_3_.

The calibration curves of AMH determination using the developed spectrofluorometric systems were plotted using FI as a function of the drug concentrations (Figure 9). The linear concentration range was found to be at 0.1–200 and 1.0–100 ng mL^−1^ for the AMH-Al(NO_3_)_3_-SDS complex. The regression equation was FI = 3.2086x + 274.31 (r = 0.9995) and FI = 2.8189x + 187.41 (r = 0.9990).

Statistical analysis of the obtained results revealed a high correlation coefficient (r) value (˃0.999) and a low standard deviation value of the intercept (S_a_) and slope (S_b_), indicating excellent linearity of the plotted curve (Table 2).

The suggested spectrofluorometric methods’ lower limits of detection (LODs) and quantification (LOQs) were calculated from the relations 3.3(S_a_/_b_) and 10 (S_a_/_b_), respectively. The LODs of AMH-Al_2_O_3_NPs-SDS and AMH-Al(NO_3_)_3_-SDS probes were calculated to be 0.04 and 0.7 ng mL^−1^. However, the LOQs were 0.1 and 1.0 ng mL^−1^ for the abovementioned systems (Table 2).

To evaluate the accuracy of the two proposed fluorescence AMH-Al_2_O_3_NPs-SDS and AMH-Al(NO_3_)_3_-SDS probes, AMH was determined in its authentic samples. The accuracy of the suggested fluorescence systems was stated as the calculated % recoveries 99.67 ± 0.34% and 99.21 ± 0.74% in the presence of Al_2_O_3_NPs and Al(NO_3_)_3_, respectively (Table 3).

The proposed FL systems were validated with respect to their precision using intra-and interday assays. The precision was calculated as the % relative standard deviation (%RSD) and was accomplished from triplicate measurements of three analytical samples. The outcomes revealed that the intraday assay of AMH using AMH-Al_2_O_3_NPs-SDS and AMH-Al(NO_3_)_3_-SDS probes were 0.20% and 0.44%, respectively. However, the results for the interday assay were 0.27%, and 0.29% for the above two systems, respectively (Table 3). The resulting precision values were less than 2, revealing good precision of the AMH-Al_2_O_3_NPs-SDS and AMH-Al(NO_3_)_3_-SDS systems.

The robustness of the AMH-Al_2_O_3_NPs-SDS and AMH-Al(NO_3_)_3_-SDS systems for the analysis of AMH was determined by performing small variations in the method parameters. The pH of the analytical samples was increased or decreased by ±1, the surfactant volume was increased or decreased by (±0.1), and the volume of Al_2_O_3_NPs or Al(NO_3_)_3_ was increased or decreased by ±0.3 mL. The FI was not significantly changed by these variations. The results were estimated as the % recoveries of 99.62 ± 0.46% and 99.24 ± 0.83% for AMH in the presence of Al_2_O_3_NPs-SDS and Al(NO_3_)_3_-SDS, respectively (Table 3).

The ruggedness of the fluorescence AMH-Al_2_O_3_NPs-SDS and AMH-Al(NO_3_)_3_-SDS systems for the assay of AMH was studied by analyzing the same samples under different conditions, such as another laboratory, analyst, or instrument. The FI was not influenced by these changes. The estimated % recoveries were 99.54 ± 0.62% and 99.26 ± 0.87% for AMH determination in the presence of the AMH-Al_2_O_3_NPs-SDS and AMH-Al(NO_3_)_3_-SDS systems, respectively.

The selectivity of the designed spectrofluorometric AMH-Al_2_O_3_NPs-SDS and AMH-Al(NO_3_)_3_-SDS systems for the analysis of AMH was evaluated using various amino acids, sugars, and inactive ingredients present in the syrup formulations that were used as additive materials in the production of the AMH syrup. Using AMH samples (1.0 ng mL^−1^), the acceptable level was computed under optimum conditions as the number of foreign species that create an error of less than 5% (Table 4). The obtained results of the selectivity levels revealed that the developed AMH-Al_2_O_3_NPs-SDS and AMH-Al(NO_3_)_3_-SDS systems displayed acceptable selectivity for the quantification of AMH.

### 2.10. Possible Effects of Al_2_O_3_NPs and Metal Chelation

**Figure 10:**
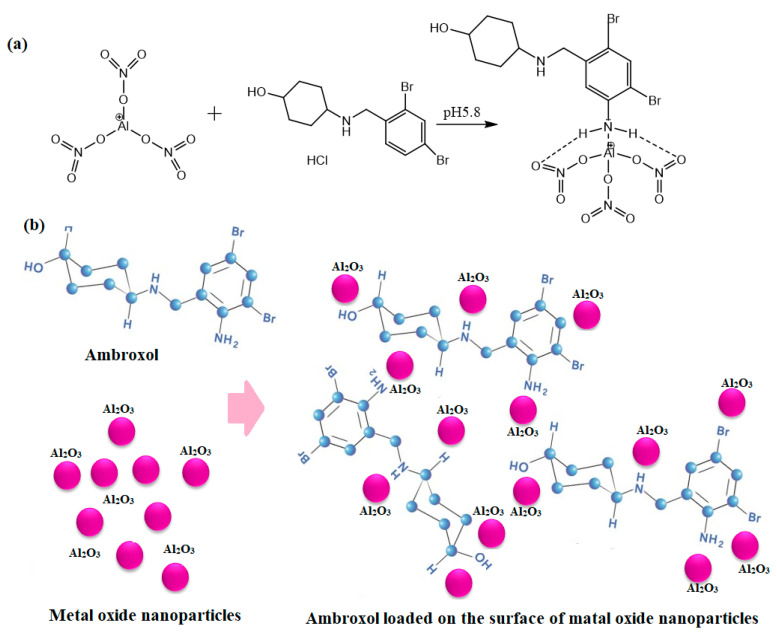
(**a**) Possible pathway for the interaction between AMH and Al(NO_3_)_3_ and (**b**) the distribution of the investigated drug on the surface of Al_2_O_3_NPs.

The interaction between the investigated AMH with Al(NO_3_)_3_ in the presence of a phosphate buffer solution of pH 5.8 can be conducted by the interaction of aminous hydrogens and oxygen atoms bonding to the aluminum action. Furthermore, the weakness of the metal–oxygen bonds can be broken to form a metal chelate surface complex (Figure 10a). The large surface-to-volume area of Al_2_O_3_NPs plays an impact role in the enhancement of the optical features of AMH and exhibits high tunable characteristics (Figure 10b).

### 2.11. Analytical Applications

The suggested spectrofluorometric AMH-Al_2_O_3_NPs-SDS and AMH-Al(NO_3_)_3_-SDS systems were used to determine AMH in its bulk powder (Table 5). The obtained results were 99.81 ± 0.41 and 99.39 ± 1.17 for AMH in the presence of Al_2_O_3_NPs-SDS and AMH-Al(NO_3_)_3_-SDS, respectively. The resulting data revealed that the presence of Al_2_O_3_NPs improved the sensitivity of the designed spectrofluorometric Al_2_O_3_NPs-SDS system due to the unique characteristics of Al_2_O_3_NPs, such as large surface areas, the wide band gap of semiconductor materials, and advanced optical and tunable mechanical properties.

The current spectrofluorometric probes were used to determine AMH in its commercial syrup, and the obtained results are summarized in Table 6. The obtained results were found to be 99.81 ± 0.41 and 99.45 ± 0.50 for AMH in the presence of Al_2_O_3_NPs-SDS and AMH-Al(NO_3_)_3_-SDS, respectively. The outcomes were mathematically assessed using the Student’s t-test and the variance ratio F-test [33], and the results were compared with those obtained using other published methodologies [10]. It was noticed that the proposed spectrofluorometric systems exhibited excellent sensitivity for the assay of AMH in the presence of spectrofluorometric Al_2_O_3_NPs-SDS and AMH-Al(NO_3_)_3_-SDS systems.

These results are explained by the enhancing potential of metal oxide nanoparticles on the collective oscillations of conduction electrons and localized surface plasmon resonances that strongly couple to light at particular wavelengths and provide the unique high optical characteristics of the materials. The high fluorescence activity of metal oxide nanoparticles was also a result of their large surface area and tunable features that were produced through the synthesis process of their nanostructures and improved the spectrofluorimetric determination of the investigated drug.

Moreover, the results from the suggested spectrofluorometric systems were compared with previously addressed analytical techniques, such as spectrophotometric, chemiluminescence, electrochemical, and chromatographic methods. The findings are presented in Table 7. The developed spectrofluorometric Al_2_O_3_NPs-SDS and AMH-Al(NO_3_)_3_-SDS systems revealed excellent sensitivity, simplicity, and cost-effectiveness and were ecofriendly. They are also more accurate and precise and do not require high technical skills when compared with other chromatographic or electrochemical techniques.

## 3. Materials and Methods

### 3.1. Chemicals

All chemicals and reagents are analytical, pure grade and applied for measurements without further purification. Methanol (99.8%) and ethanol (96.9%) were purchased from Sigma-Aldrich, Hamburg, Germany. Acetonitrile (99.9%), acetic acid, sodium acetate (99.0%), sodium dihydrogen phosphate (98.0%), boric acid (99.8%), aluminum nitrate nonahydrate (Al (NO_3_)_3_. 9 H_2_O (99.9%), sodium tetraborate (99.9%), and cetylpyridium chloride (CPC, 99.9%) were supplied by BDH Ltd., Pool, UK. However, sodium dodecyl sulfate (SDS, 99.5%), sodium hydroxide (95.0%), and Triton X-100 (99.0%) were acquired from WinLab, East Midlands, UK. Pure grade ambroxol hydrochloride (AMH) was kindly supplied by Tabuk Pharmaceutical Co., Tabuk, Saudi Arabia. The pharmaceutical preparation MUCOSOLVAN^®^ 0.3% *w*/*v* syrup was purchased from local drug stores (Riyadh, Saudi Arabia).

### 3.2. Plant Material

In the current study, dried *Lavandula spica* flowers of Saudi Arabian origin were used. The moisture content of the flowers was detected by drying to a constant mass at 100 ± 5 °C and was less than 2%. The flowers were grounded in a grinder (GT11, Tefal, Rumilly, France) and stored at room temperature in a tight container until use.

### 3.3. Instruments

An RF-5301pc luminescence spectrometer (Shimadzu, Kyoto, Japan) was used for all spectrofluorometric measurements. The slit widths for both the excitation and emission monochromators were set at 5 nm, which was used to conduct all spectrofluorometric measurements. The outcomes were controlled by a PC and data gathering was performed using FI Winlab software (version 4.00.03). A pH-meter Metrohm model 744 (Metrohm Co., Herisau, Switzerland) was used to regulate the pH of the samples under investigation. Throughout the experiment, deionized water was utilized, and it was obtained via Lonenaustausscher, SG, Germany. Several techniques were applied to reveal the synthesis of Al_2_O_3_NPs, including Shimadzu-spectrophotometer-2600i (Kyoto, Japan), Perkin-Elmer-spectrometer (Waltham, MA, USA), 6000-X-ray Shimadzu diffractometer (Kyoto, Japan), JSM-7610F scanning electron microscope (SEM, Tokyo, Japan), energy-dispersive X-Ray spectrometer (EDX, Tokyo, Japan), transmission electron microscope (TEM, JEOL Ltd., Tokyo, Japan).

### 3.4. Preparation of Plant Extract 

The preparation of *Lavandula spica* flowers was prepared using a previously reported method with slight modification [38]. The dried powder of *Lavandula spica* flowers (1.0 g) was mixed with 50 mL of deionized water and heated under constant magnetic stirring for 30 min at 60 °C. The resulting mixture was then centrifuged using 3500 rpm and filtered using Whatman filter paper no. 40. The resulting filtrate was cooled and stored in clean containers until further use in the biogenic synthesis of Al_2_O_3_NPs.

### 3.5. Preparation of Al_2_O_3_NPs Using Lavandula spica Flower Extract

**Scheme 1:**
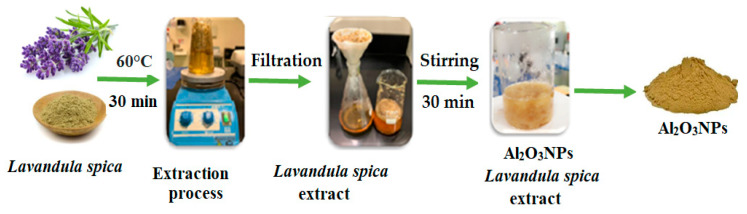
Illustration of the green synthesis of Al_2_O_3_NPs using *Lavandula spica* flower extract.

The green preparation of Al_2_O_3_NPs was conducted using the previous literature [39] with slight changes. The preparation procedure was started by mixing 20 mL of *Lavandula spica* flower extract with 100 mL of aluminum nitrate nonahydrate (2.0 mol L^−1^) and left under continuous agitation for 30 min. Yellowish-brown Al_2_O_3_NPs were formed. The formed nanoparticles were filtered after being centrifuged at 3500 rpm for 5 min, and the resulting nanomaterials were collected, dried, and kept in a clean container for further studies. The stock solution of Al_2_O_3_NPs was obtained by sonicating 0.1 g of the pre-synthesized powder in 100 mL deionized water for 10 min and stored in the refrigerator at 4 °C (Scheme 1).

### 3.6. Characterization of the Synthesized Al_2_O_3_NPs

The synthesized Al_2_O_3_NPs were confirmed by several analytical techniques, such as spectroscopic and microscopic. Among these techniques were ultraviolet–visible spectroscopy (UV-vis), Fourier-transform infrared spectroscopy (FT-IR), X-ray powder diffraction spectroscopy (XRD), and scanning and transmission electron microscopy (SEM and TEM). Furthermore, the elemental content and mapping were performed using the energy-dispersive X-ray method (EDX).

### 3.7. Preparation of Analytical Reagents

Three different buffer solutions (acetate, phosphate, and borate) were prepared to cover a pH range from 3 to 9.3. Acetate buffer solution (0.1 mol L^−1^) in a pH range of 3.0–5.6 was prepared by mixing equivalent volumes of 0.1 mol L^−1^ acetic acid and 0.1 mol L^−1^ sodium acetate solution to obtain the desired pH. Meanwhile, phosphate buffer (0.2 mol L^−1^) in a pH range of 5.8–8 was prepared by adding equivalent amounts of 0.2 mol L^−1^ of sodium dihydrogen to 0.2 mol L^−1^ sodium hydroxide and adjusted to gain suitable pH values. However, borate buffer (0.1 mol L^−1^) of pH 8–9.3 was prepared by adding equivalent quantities each of 0.1 mol L^−1^ boric acid and 0.1 mol L^−1^ sodium tetraborate, and the pH was adjusted to reach the desired pH range.

Different surface-active agents, including 0.01 mol L^−1^ of sodium dodecyl sulfate (SDS), cetylpyridinium chloride (CPC), and Triton X-100, were prepared by dissolving 0.28 g, 0.33 g, and 0.15 mL in 100 mL deionized water of the abovementioned surfactants in a 100 mL volumetric flask, respectively. An aqueous solution of 0.01 mol L^−1^ Al(NO_3_)_3_.9H_2_O was prepared by dissolving 0.37 g of solid material in 100 mL deionized water.

### 3.8. Preparation of Standard AMH and Commercial Syrup Samples

A stock-standard solution of 100 µg mL^−1^ AMH was obtained by dissolving an accurate quantity of 10 mg of AMH in 100 mL deionized water. The prepared solution was stored in an amber glass bottle and kept aside in a dark place for further use. The working analytical samples in a concentration range of 0.001–0.1 µg mL^−1^ was serially diluted using deionized water. However, to prepare 1.0 µg mL^−1^ of MUCOSOLVAN 0.3% *w*/*v* syrup, 0.017 mL of syrup was mixed with 100 mL deionized water. The working analytical solutions in the range of 0.001–0.08 µg mL^−1^ was prepared by serial dilution using deionized water.

### 3.9. General Procedure for AMH Determination

Probe 1: At ambient temperature, the FI for AMH assay was investigated in the presence of Al_2_O_3_NPs and SDS (1.0% *w*/*v*). The analytical samples of the test drug using 10 mL volumetric flasks were analyzed to determine AMH in its pure sample and syrup. The FI was measured at λ_ex_ 244 and λ_em_ 420 nm using 1.5 mL of Al_2_O_3_NPs, 0.5 mL of SDS, and 2.0 mL of phosphate buffer (0.2 mol L^−1^, pH 5.8). The linearity of the drug detection was expressed as calibration graphs, and a regression equation was derived to evaluate the unknown concentration of the examined drug.

Probe 2: To plot the calibration graph of the suggested method, a final AMH concentration in the range of 0.001–0.1 µg mL^−1^ was prepared. Approximately 1.5 mL of 0.01 mol L^−1^ Al(NO_3_)_3_.9H_2_O solution, 1.5 mL of 0.01 mol L^−1^ SDS, and 2.0 mL of 0.2 mol L^−1^ phosphate buffer (pH 5.8) was mixed with an equivalent volume of AMH drug in 10 mL volumetric flasks to obtain the required concentrations. Then, the solution was mixed well and completed to volume with deionized water. The fluorescence intensity (FI) of each sample was recorded at emission wavelength λ_em_ 369 nm after exaction wavelength at λ_ex_ 244 nm.

## 4. Conclusions

This research study demonstrated accurate and exact fluorescent probes based on metal oxide nanoparticles (Al_2_O_3_) and metal-chelating agents (Al(NO_3_)_3_) for AMH measurement in bulk and commercial syrup. The work relied on the extraordinary optical properties of the developed spectrofluorometric AMH-Al_2_O_3_NPs-SDS and AMH-Al(NO_3_)_3_-SDS systems, which amplified the fluorescence intensity of the selected medication. The data from the recommended fluorescence probes revealed that Al_2_O_3_NPs and Al (NO_3_)_3_ had good catalytic activity and that the rise in fluorescence was precisely proportionate to the increase in drug concentrations. The findings showed excellent linearity over wide concentration ranges, and the two fluorescence probes, the AMH-Al_2_O_3_NPs-SDS and AMH-Al(NO_3_)_3_-SDS systems, can be potentially applied for the determination of AMH in its authentic powder and commercial syrup, with mean percentage recoveries of 99.81 ± 0.41, 99.39 ± 1.17 and 99.81 ± 0.41, 99.45 ± 0.50, respectively. The resulting data were statistically analyzed, and the results agreed well with those reported from previously published methods. The proposed fluorescence methods exhibited great catalytic activity and sensitivity due to the amplification of surface plasmon resonance and collective oscillations of conduction electrons that linked with light, resulting in their strong optical properties.

## Figures and Tables

**Table 1:** Optimized conditions for the determination of AMH using the proposed spectrofluorometric methods in the presence of Al_2_O_3_NPs and metal-chelating agent Al (NO_3_)_3_.

Parameter	Studies Range	AMH	
		Al_2_O_3_NPs	Al(NO_3_)_3_
λ_ex/em_ (nm)	220–800	260/460	244/369
Buffer type	Acetate, phosphate, borate	Phosphate	Phosphate
Buffer pH	3–9.3	5.8	5.8
Buffer volume (mL)	0.5–5	1.5	2.0
Al_2_O_3_NPs and Al(NO_3_)_3_ volume	0.5–5	1.5	2.0
Surfactant type	SDS, CPC, and CMC	SDS	SDS
Surfactant volume (mL)	0.5–3	1.0	2.0
Time (min)	1–10	2	2

**Table 2:** Response data obtained from the determination of AMH using the proposed spectrofluorometric Al_2_O_3_NPs-SDS and Al(NO_3_)_3_-SDS systems.

Parameter	AMH-Al_2_O_3_NPs-SDS	AMH-Al(NO_3_)_3_-SDS
Concentration range, ng mL^−1^	0.1–200	1.0–100
Slope	3.2086	2.8189
Intercept	274.31	187.41
Correlation coefficient (r)	0.9995	0.9990
Standard deviation of intercept (S_a_)	3.113812	4.673863
Standard deviation of slope (S_b_)	0.033849	0.088873
LOD, (ng mL^−1^)	0.04	0.7
LOQ, (ng mL^−1^)	0.1	1.0

**Table 3:** Results of validation calculated from the determination of AMH in authentic samples using the proposed spectrofluorometric AMH-Al_2_O_3_NPs-SDS and AMH-Al(NO_3_)_3_-SDS systems.

Samples	Accuracy (n = 9)	Intraday (n = 3)	Interday (n = 3)	Repeatability (RSD %, n = 6)	Robustness	Ruggedness
AMH-Al_2_O_3_NPs-SDS	99.21 ± 0.74	0.20%	0.27%	0.53%	99.62 ± 0.46	99.54 ± 0.62
Al(NO_3_)_3_-SDS	99.67 ± 0.34	0.44%	0.29%	0.82%	99.24 ± 0.83	99.26 ± 0.87

**Table 4:** Influence of foreign substances on the analysis of AMH using the suggested spectrofluorometric Al_2_O_3_NPs-SDS and AMH-Al(NO_3_)_3_-SDS systems.

Interference	Tolerable Value	
	**AMH-Al_2_O_3_NPs-SDS**	**AMH-Al(NO_3_)_3_-SDS**
Glycerol	250	320
Benzoic acid	360	405
Vanilla aroma	400	390
Hydroxyethyl cellulose	520	480
Lactose	190	240
Glucose	210	310
Glycine	640	570
Histidine	160	250
L-valine	240	390
Leucine	270	430

**Table 5:** Results of AMH assay using the suggested spectrofluorometric Al_2_O_3_NPs-SDS and AMH-Al(NO_3_)_3_-SDS systems.

Sample	Taken (ng mL^−1^)	Found Range (ng mL^−1^)	% Recovery	Mean ± SD	n	Variance	% SE	% RSD
AMH-Al_2_O_3_NPs-SDS	0.1–200	0.1–199.85	98.99–100.00	99.81 ± 0.41	6	0.17	0.16	0.41
AMH-Al(NO_3_)_3_-SDS	1.0–100	1.0–100	97.00–99.98	99.39 ± 1.17	6	1.37	0.47	1.17

**Table 6:** Assay of AMH in its commercial syrup applying the proposed Al_2_O_3_NPs-SDS and Al(NO_3_)_3_-SDS spectrofluorometric system.

Sample	Taken (ng mL^−1^)	Found (ng mL^−1^)	% Recovery	Mean ± SD	n	Var.	% SE	% RSD	Ref. Method [10]	*t*-Test (2.228) *	F-Test (5.05) *
AMH-Al_2_O_3_NPs-SDS	0.1–200	0.1–200	0.098–200.2	99.81 ± 0.41	6	0.17	0.16	0.41	99.57 ± 0.52	0.909	1.59
AMH-Al(NO_3_)_3_-SDS	1.0–100	0.95–99.99	98.78–99.83	99.45 ± 0.50	6	0.25	0.10	0.50		0.516	1.08

* Tabulated *t*-test and F-test values at *p* < 0.05.

**Table 7:** A comparison between the current results from the assay of AMH applying the suggested spectrofluorometric Al_2_O_3_NPs-SDS and AMH-Al(NO_3_)_3_-SDS spectrofluorometric systems and the previously reported analytical techniques.

Analytical Technique	Reagent	Linearity	LOD	Reference
Spectrophotometry	AMH, based on first-order derivative	7.5–45 μg mL^−1^		[34]
Chemiluminescence	AMH, redox reaction of Ce(IV)-sodium sulfite in H_2_SO_4_	7.0 × 10^−8^–1 × 10^−6^ g mL^−1^	3 × 10^−8^ g mL^−1^	[35]
Electrochemical	AMH, electrodeposited ZnONPs on carboxylated CNTs modified glassy carbon electrode	1–120 μmol L^−1^	0.02 μmol L^−1^	[36]
Chromatography	AMH, RP-HPLC method, buffer (pH 3.5) and acetonitrile (40:60% *v*/*v*)	7.5–45 μg mL^−1^		[37]
Proposed method	Spectrofluorometric measurement in the presence of Al_2_O_3_NPs and Al(NO_3_)_3_	0.1–200 ng mL^−1^		AMH-Al_2_O_3_NPs
		1–100 ng mL^−1^		AMH-Al(NO_3_)_3_

## Data Availability

All data have been included in the text.

## Sample Availability

Samples of the compounds are available from the authors.

## References

[1] Migliori G.B., Marx F.M., Ambrosino N., Zampogna E., Schaaf H.S., van der Zalm M.M., Allwood B., Byrne A.L., Mortimer K., Wallis R.S. (2021). Clinical standards for the assessment, management and rehabilitation of post-TB lung disease. Int. J. Tubercul. Lung Dis..

[2] Dippenaar A., Goossens S.N., Grobbelaar M., Oostvogels S., Cuypers B., Laukens K., Meehan C.J., Warren R.M., Van Rie A. (2022). Nanopore sequencing for mycobacterium tuberculosis, A critical review of the literature, new developments, and future opportunities. Eur. J. Clin. Microbiol..

[3] Md S., Abdullah S.T., Alhakamy N.A., Bani-Jaber A., Radhakrishnan A.K., Karim S., Shahzad N., Gabr G.A., Alamoudi A.J., Rizg W.Y. (2021). Ambroxol hydrochloride loaded gastro-retentive nanosuspension gels potentiate anticancer activity in lung cancer (A549) cells. Gels.

[4] Panchale W.A., Badukle N.A., Sabhadinde A.F., Bakal R.L., Manwar J.V. (2020). Concurrent analysis of ambroxol HCl and salbutamol sulphate from tablet formulation by RP-HPLC. GSC Biol. Pharm. Sci..

[5] El-Sayed H.M., Hashem H. (2020). Quality by design strategy for simultaneous HPLC determination of bromhexine HCl and its metabolite ambroxol HCl in dosage forms and plasma. Chromatographia.

[6] El-Sayed A., Elmansi H., Shalan S., Eid M. (2022). Facile approaches for determination of Bromhexine Hydrochloride and its active metabolite Ambroxol Hydrochloride using Eosin Y. Annales Pharmaceutiques Françaises.

[7] Prabu S.L., Shirwaikar A.A., Shirwaikar A., Kumar C.D., Kumar G.A. (2008). Simultaneous UV spectrophotometric estimation of ambroxol hydrochloride and levocetirizine dihydrochloride. Ind. J. Pharm. Sci..

[8] Rele Rajan V., Gurav Pankaj J. (2012). Simple Spectrophotometric methods for determination of Ambroxol Hydrochloride from pharmaceutical formulation. Int.J. PharmTech Res..

[9] Shah P.S., Patel K.G., Shah P.A., Gandhi T.R. (2020). UV-spectrophotometry-assisted chemometric methods for simultaneous determination of ambroxol hydrochloride and doxofylline in pharmaceutical formulation. J. Chem. Metrol..

[10] Levent A., Yardım Y., Şentürk Z. (2014). Electrochemical performance of boron-doped diamond electrode in surfactant-containing media for ambroxol determination. Sens. Actuators B Chem..

[11] Liu J., Sui X. (2021). Hospital wireless sensor network coverage and ambroxol hydrochloride in the treatment of mycoplasma pneumonia in children. Microprocess Microsyst..

[12] Dong M.W. (2013). The Essence of Modern HPLC: Advantages, Limitations, Fundamentals, and Opportunities. LCGC N. Am..

[13] Refat M.S., Saad H.A., Gobouri A.A., Alsawat M., Adam A.M., Shakya S., Gaber A., Alsuhaibani A.M., El-Megharbel S.M. (2022). Synthesis and spectroscopic characterizations of nanostructured charge transfer complexes associated between moxifloxacin drug donor and metal chloride acceptors as a catalytic agent in a recycling of wastewater. J. Mol. Liq..

[14] Mishra R.K., Ha S.K., Verma K., Tiwari S.K. (2018). Recent progress in selected bio-nanomaterials and their engineering applications: An overview. J. Sci. Adv. Mater. Devices.

[15] Mahmoud W.H., Mohamed G.G., Elsawy H.A., Radwan M.A. (2018). Metal complexes of novel Schiff base derived from the condensation of 2-quinoline carboxaldehyde and ambroxol drug with some transition metal ions. Appl. Organomet. Chem..

[16] Pipattanaporn P., Pansiri P., Kumpeerakij P., Yaemphutchong S., Siri-apai P., Suetrong N., Chansaenpak K., Singkammo S., Kanjanaboos P., Hanlumyuang Y. (2022). Effect of triethanolamine chelating agent on crystallinities, phase purities, and optical properties of zinc aluminate spinel synthesized by thermal decomposition. Ceram. Int..

[17] Prozorovska L., Baker B.A., Laibinis P.E., Jennings G.K. (2021). Surface-Initiated, Catechol-Containing Polymer Films for Effective Chelation of Aluminum Ions. Langmuir.

[18] Ziva A.Z., Suryana Y.K., Kurniadianti Y.S., Nandiyanto A.B., Kurniawan T. (2021). Recent progress on the production of aluminum oxide (Al_2_O_3_) nanoparticles: A review. MESI.

[19] Meng L.Y., Jiang W., Piao W., Meng W. (2016). Effect of bio-template on the properties of SiO_2_/Al_2_O_3_ composites for drug delivery. J. Ind. Eng. Chem..

[20] Sani O.N., Yazdani M., Taghavi M. (2019). Catalytic ozonation of ciprofloxacin using γ-Al_2_O_3_ nanoparticles in synthetic and real wastewaters. JWPE.

[21] Hassanpour P., Panahi Y., Ebrahimi-Kalan A., Akbarzadeh A., Davaran S., Nasibova A.N., Khalilov R., Kavetskyy T. (2018). Biomedical applications of aluminium oxide nanoparticles. Micro Nano Lett..

[22] Tulinski M., Jurczyk M. (2017). Nanomaterials synthesis methods. Metrology and Standardization of Nanotechnology: Protocols and Industrial Innovations.

[23] Shamaila S., Sajjad A.K., Farooqi S.A., Jabeen N., Majeed S., Farooq I. (2016). Advancements in nanoparticle fabrication by hazard free eco-friendly green routes. Appl. Mater. Today.

[24] Sumesh K.R., Kanthavel K. (2019). Green synthesis of aluminium oxide nanoparticles and its applications in mechanical and thermal stability of hybrid natural composites. J. Polym. Environ..

[25] Kurkin V.A., Lamrini M., Klochkov S.G. (2008). Lavandoside from *Lavandula spica* flowers. Chem. Natural Comnds..

[26] Badr M.M., Badawy M.E., Taktak N.E. (2021). Characterization, antimicrobial activity, and antioxidant activity of the nanoemulsions of *Lavandula spica* essential oil and its main monoterpenes. J. Drug Deliv. Sci. Technol..

[27] Ahmed A.S., Singla M.L., Tabassum S., Naqvi A.H., Azam A. (2011). Band gap narrowing and fluorescence properties of nickel doped SnO_2_ nanoparticles. J. Lumin..

[28] Prashanth P.A., Raveendra R.S., Hari Krishna R., Ananda S., Bhagya N.P., Nagabhushana B.M., Lingaraju K., Raja N.H. (2015). Synthesis, characterizations, antibacterial and photoluminescence studies of solution combustion-derived α-Al_2_O_3_ nanoparticles. J. Asian Ceram. Soc..

[29] Özsevinç A., Alkan C. (2023). Polyurethane shell medicinal lavender release microcapsules for textile materials: An environmentally friendly preparation. Ind. Crops Prod..

[30] Tanna J.A., Gomaji C.R., Gandhare N.V., Juneja H.D. (2016). Alumina nanoparticles: A new and reusable catalyst for synthesis of dihydropyrimidinones derivatives. Adv. Mater. Lett..

[31] Mulon J.B., Destandau É., Alain V., Bardez É. (2005). How can aluminium (III) generate fluorescence?. J. Inorg. Biochem..

[32] ICH Q2 (R1): Validation of analytical procedures: Text and methodology. Proceedings of the International Conference on Harmonization.

[33] Miller J.C., Miller. J.N. (1993). Statistics for Analytical Chemistry.

[34] Panchale W.A., Bakal R.L. (2021). First-order derivative spectrophotometric estimation of gemifloxacin mesylate and ambroxol HCl in tablet dosage form. GSC Biol..

[35] Xiong F.M., Tang Y.H., Sun S.J., Wang N.N. (2006). Determination of ambroxol using flow-injection chemiluminescence method. J. Chin. Pharm. Sci..

[36] Li L. (2021). Electrochemical Methods for Quantitative Estimation of Ambroxol Hydrochloride in drug for the Treatment of Asthmatic Bronchitis. Int. J. Electrochem. Sci..

[37] Akram N.M., Gopal N.M., Balakrishna A., Reddy N.B., Sravya G., Zyryanov G.V. (2022). RP-HPLC method for the simultaneous analysis of ambroxol hydrochloride and nitazoxanide in API and tablet dosage form. AIP Conf Proc..

[38] Guo X., Wang P. (2020). Aroma Characteristics of Lavender Extract and Essential Oil from Lavandula angustifolia Mill. Molecules.

[39] Manikandan V., Jayanthi P., Priyadharsan A., Vijayaprathap E., Anbarasan P.M., Velmurugan P. (2019). Green synthesis of pH-responsive Al_2_O_3_ nanoparticles: Application to rapid removal of nitrate ions with enhanced antibacterial activity. J. Photochem. Photobiol. B.

